# *In vitro* remineralizing effect of two mouthwashes on the enamel of demineralized permanent molars

**DOI:** 10.21142/2523-2754-1402-2026-289

**Published:** 2026-04-04

**Authors:** Daniela Soledad Rodríguez-Chávez, Yuri Freddy Curo-Valdivia, Carol Ximena Portales-Carbonel

**Affiliations:** 1 Stomatology Study Program, Faculty of Medicine, Antenor Orrego Private University. Trujillo, Peru. drodriguezc15@upao.edu.pe mat2192@hotmail.com carol-ximena21@hotmail.com Universidad Privada Antenor Orrego Stomatology Study Program Faculty of Medicine Antenor Orrego Private University Trujillo Peru drodriguezc15@upao.edu.pe mat2192@hotmail.com carol-ximena21@hotmail.com

**Keywords:** dental remineralization, xylitol, fluoride, mouthwashes, remineralización dental, xilitol, flúor, antisépticos bucales

## Abstract

**Objective:**

To compare the in vitro remineralizing effect of two mouthwashes on the enamel of demineralized permanent molars.

**Materials and methods:**

The study was experimental and conducted *in vitro*. Twenty healthy third molars were used. They were randomly divided into two groups (xylitol mouthwash vs. fluoride mouthwash). After demineralizing the tooth crowns with 37% orthophosphoric acid, they were immersed in the mouthwash treatments at 37 °C for 15, 30, and 45 days, with mouthwash replacement every 3 days. The degree of remineralization was evaluated in 150-250 µm sections under a polarized light microscope (EUROTECH), using the following scale: 0 (no change), 1 (superficial), 2 (sub superficial), and 3 (deep). The Mann-Whitney U test, Friedman test, and Holm post hoc test were used (p < 0.05).

**Results:**

The fluoride mouthwash showed significantly higher remineralization Grades than xylitol at all time intervals: at 15 days, 60% vs. 30% grade 1 (p = 0.481); at 30 days, 20% grade 1, 60% grade 2, and 20% grade 3 vs. 50% grade 1 and 10% grade 2 (p = 0.052); and at 45 days, 60% grade 3, 40% grade 2 vs. 30% grade 1 and 30% grade 2 (p = 0.043). Both treatments showed significant evolution over time (p = 0.001), but only fluoride reached its maximum effect at 45 days.

**Conclusion:**

Both mouthwashes showed a remineralizing effect on demineralized enamel; however, fluoride had a significantly superior effect.

## RESUMEN

**Objetivo:**

Comparar el efecto remineralizante *in vitro* de dos colutorios en el esmalte de molares permanentes desmineralizados.

**Materiales y métodos:**

El estudio fue experimental *in vitro*. Se utilizaron 20 terceras molares sanas, divididas aleatoriamente en dos grupos (colutorio con xilitol vs. colutorio con fluoruro). Tras desmineralizar las coronas de los dientes con ácido ortofosfórico al 37%, estas se sumergieron en los colutorios a 37 °C durante 15, 30 y 45 días, con recambio cada 3 días. El grado de remineralización se evaluó en cortes de 150-250 µm bajo microscopio de luz polarizada (Eurotech), usando la escala: 0 (sin cambio), 1 (superficial), 2 (subsuperficial) y 3 (profundo). Se emplearon las pruebas de U de Mann-Whitney, Friedman y *post hoc* de Holm (p < 0,05).

**Resultados:**

El colutorio con fluoruro mostró una remineralización significativamente mayor que el xilitol en todos los tiempos: a los 15 días, 60% vs. 30% grado 1 (p = 0,481); a los 30 días, 20% grado 1, 60% grado 2 y 20% grado 3 vs. 50% grado 1 y 10% grado 2 (p = 0,052); y a los 45 días, 60% grado 3, 40% grado 2 vs. 30% grado 1 y 30% grado 2 (p = 0,043). Ambos tratamientos mostraron evolución significativa con el tiempo (p = 0,001), pero solo el fluoruro alcanzó su máximo efecto a los 45 días.

**Conclusión:**

Ambos colutorios mostraron un efecto remineralizante en el esmalte desmineralizado; sin embargo, el fluoruro fue el que ejerció un efecto significativamente superior.

## INTRODUCTION

Early diagnosis of incipient dental lesions can open a new era in preventive dentistry, as it will allow remineralization to be an effective form of treatment ^1^. Among the numerous preventive strategies recommended for combating tooth decay, fluoride application is a widely accepted and extremely effective treatment modality.^2^^,^^3^ There is, however, growing interest in fluoride-free options, such as xylitol, which has been associated with increased mineralization in dental tissues. Several studies, both clinical and laboratory, have documented the remineralization of dental caries by regular consumption of chewing gum containing xylitol ^4^^,^^5^.

Dental caries is known to be a chronic, non-communicable disease that develops when cariogenic bacteria adhere to teeth and metabolize sugars, thereby producing acid that demineralizes the tooth structure over the course of time ^6^. Increased acidity in the oral cavity leads to demineralization of hydroxyapatite crystals in the tooth enamel, and dispersion of calcium and phosphate ions ^7^. The above-mentioned conditions produce an imbalance in the cyclical process of demineralization and remineralization of the dental structure, which is further influenced by bacterial biofilms. This imbalance occurs due to the acidic byproducts generated by these bacteria, which cause a significant decrease in pH. When the drop in pH exceeds the critical limit for the enamel this prevents it from returning to its initial levels in time. This in turn leads to the (demineralizing and remineralizing) process not being modulated by the neutralizing action of saliva ^8^.

When repeated mineral dissolution events are produced and subsurface mineral loss exceeds gain over a long period, the first clinical sign of (caries) disease is a white spot lesion. The progression of this white spot and the subsequent development of cavitated caries can be prevented, reversed, or halted by controlling the caries process ^9^.

Fluoride present in the biofilm due to regular topical exposure and surrounding carbonated apatite crystals (enamel crystals) effectively inhibits tooth demineralization. It also enhances tooth remineralization by accelerating the growth of fluorapatite crystals in the partially demineralized subsurface crystals of the carious lesion ^10^. In contrast, xylitol, a naturally occurring five-carbon sugar polyol, is a white crystalline carbohydrate that has been known for a century. Over the last 40 years, it has been extensively researched for its effect on dental caries ^11^.

Xylitol, used as an artificial sweetener in foods, cannot be metabolized by oral bacteria, which contributes to the prevention of cavities. Although the effects of xylitol chewing gum on caries have been evaluated, there is less literature on its effectiveness as a mouthwash ^12^. The objective of this study was to make a comparison between two mouthwashes, one with xylitol and one with sodium fluoride with respect to their in vitro remineralizing effect on the enamel of demineralized permanent molars.

## MATERIALS AND METHODS

### Study design

An *in vitro* experimental design study was conducted in compliance with the consent of the Institutional Ethics Committee of the Antenor Orrego Private University. BIOETHICS COMMITTEE RESOLUTION No. 01328-2024-UPAO.

### Sample assignment and selection criteria

The traditional formula for calculating sample size for comparing two proportions was used, considering a significance of 5% and a statistical power of 80% which was, based on the expected proportion of 0.87 and 0.26 in the groups, respectively, a sample size of 10 was estimated for each group, based on the results of the pilot test. Therefore, 20 healthy third molars, recently extracted, for orthodontic or medical reasons (not due to caries) were collected, from the Stomatology Program of the Antenor Orrego Private University. The specimens were preserved in deionized water, until time of use. Teeth with deteriorated or eroded enamel, fluorosis, enamel hypoplasia, white lesions, or those with previous restorations, which could interfere with the study results, were excluded. The teeth selected were randomly divided into two groups: A (Xylitol Mouthwash) B (Fluoride Mouthwash). 

### Sample preparation and processing

The crowns and roots of the teeth were transversally sectioned to separate them from each other, using a diamond disc. The crowns were embedded in acrylic blocks, leaving their buccal surfaces exposed. Demineralization was initiated with application of an etching agent, 37% orthophosphoric acid Condac37 FGM) to specimens for 20 seconds, then they were rinsed with running water. The crowns were then sectioned in a vestibulo-lingual direction and divided into two equal parts. After this, 2 subgroups (left halves and right halves) (A and B) were established from the groups. After the demineralization process, the left half of each tooth (Subgroup A) was immersed in the remineralizing agents: Xylitol-based mouthwash (Lacer Nature®) and in Fluoride-based mouthwash (Listerine® Total Care) in sterile plastic bottles. These were used for the collection of biological fluids the right halves (Subgroups B) were submitted to the demineralization process only, and were subsequently immersed in deionized water. The samples were placed in a domestic oven to achieve a constant temperature of 37°C, and the mouthwashes were replaced f every third day. On completion of the exposure time intervals (15, 30 and 45 days), according to the Groups, longitudinal sections of 150 um to 250 um thickness were performed with a diamond disc. After sectioning, the samples were mounted on sample holders and a drop of 1.47 IR glycerol solution was applied to them. Then they were observed under a Eurotech polarized light microscope. To assess the degree of the remineralizing effect on the dentition, the following scale as used in the study by Cobos et al. ^13^ was used:

“0”: No change.

“1”: Superficial change

“2”: Subsurface change

“3”: Deep Chan

Prior to assessment of the remineralizing effect, calibration for the correct use of the polarized light microscope (Eurotech) was conducted by the chief investigator, during the pilot study. This was applied to a total of 6 third molars (3 per group) for 7 days, in accordance with the criteria that were previously defined. Intra-rater reliability was estimated based on Cohen's Kappa coefficient, which obtained a value of 1. Likewise, inter-rater reliability between the chief researcher and an expert was also estimated, in which a value of 0.714 was obtained.

### Statistical analysis

The data were processed using the free statistical software program RStudio (https://www.r-project.org/). Since an ordinal type of result variable was obtained, non-parametric tests based on ranges that respect the order of the categories were used, without assuming equality of intervals or normality of distribution ^14^. For this reason, for comparisons between two independent groups at each time interval (Xylitol vs Control; Fluoride vs Control; Xylitol vs Fluoride) the Mann-Whitney U test was applied. Whereas for intragroup comparisons over the time intervals of 15, 30 and 45 days the Friedman test for repeated measures was used. Pairwise comparisons were evaluated using the Holmes' post hoc test, and exact values of statistical significance were reported. Data were presented as absolute and relative frequencies according to the degree of remineralization. 

The significance level of α = 0.05 was adopted (two-tailed tests).

## RESULTS

Table 1 presents comparison of the degree of remineralization in the enamel of demineralized permanent molars treated with xylitol and fluoride mouthwashes versus their respective controls at time intervals of 15, 30 and 45 days. After 15 days, the xylitol group showed 30% of specimens with superficial change (Grade 1) compared to 10% of the control, with no significant differences (p = 0.481). At 30 days, xylitol concentrations reached 50% in Grade 1 and 10% in Grade 2 (subsurface), while the control remained unchanged at 90% (Grade 0); however, no significant differences were found (p = 0.052). After 45 days, statistically significant differences were presented (p = 0.043), with 30% of the specimens with xylitol showing Grade 1 and another 30% with Grade 2, in contrast with 90% without change in the Control Group. Whereas, the fluoride mouthwash specimens showed a more pronounced effect: at 15 days, 60% had Grade 1 and 20% had Grade 2 (p = 0.002); at 30 days, 20% had Grade 1, 60% had Grade 2, and 20% had Grade 3 (deep) (p < 0.001). At 45 days, 10% had Grade 1, 40% had Grade 2, and 60% had Grade 3, compared with 90% with no change in the Control Group (p < 0.001).

**Table 1:** . Degree of in vitro remineralization obtained with use of mouthwashes with Xylitol and Fluoride, respectively, on the enamel of demineralized permanent molars

Remineralization	15 days				30 days				45 days			
	Xylitol		Control		Xylitol		Control		Xylitol		Control	
	n	%	N	%	n	%	N	%	n	%	n	%
Grade 0	7	70	9	90	4	40	9	90	4	40	9	90
Grade 1	3	30	1	10	5	50	1	10	3	30	1	10
Grade 2	0	0	0	0	1	10	0	0	3	30	0	0
Grade 3	0	0	0	0	0	0	0	0	0	0	0	0
p-value*	0.481				0.052				0.043**			
	Fluoride		Control		Fluoride		Control		Fluoride		Control	
Grade 0	2	20	10	100	0	0	9	90	0	0	9	90
Grade 1	6	60	0	0	2	20	1	10	0	0	1	10
Grade 2	2	20	0	0	6	60	0	0	4	40	0	0
Grade 3	0	0	0	0	2	20	0	0	6	60	0	0
p-value*	0.002**				<0.001**				<0.001**			

*Nonparametric Mann Whitney U test. Leve of Confidence 95%

**Statistically significant differences; *p* < 0.05

Table 2 shows the direct comparison of the degree of remineralization in the samples treated with xylitol mouthwash vs. fluoride mouthwash at each exposure time interval. At 15 days, fluoride achieved 60% Grade 1 and 20% Grade 2, remineralization in the samples compared with 30% Grade 1 obtained with xylitol (p = 0.035). At 30 days, 20% of fluoride samples reached Grade 1, 60% G2 and 20% Grade 3, while xylitol only achieved 50% Grade 1 and 10% Grade 2 (p = 0.002) remineralization in the samples. Finally, at 45 days, fluoride obtained 60% of specimens with deep remineralization (Grade 3), while xylitol samples were distributed in 30% Grade 1 and 30% Grade 2 (p < 0.001), confirming a significantly superior effect of fluoride at all tie intervals. In Graphs 1 and 2, all the stages of remineralization of enamel samples exposed to Xylitol and Fluoride oral mouthwashes, respectively may be visualized.

**Table 2:** Comparison between mouthwashes with Xylitol and Fluoride with regard to the in vitro remineralizing effect on demineralized permanent molar enamel

Remineralization	15 days				30 days				45 days			
	Xylitol		Fluoride		Xylitol		Fluoride		Xylitol			Fluoride	
	n	%	n	%	n	%	N	%	N	%	n	%
Grade 0	7	70	2	20	4	40	0	0	4	40	0	0
Grade 1	3	30	6	60	5	50	2	20	3	30	0	0
Grade 2	0	0	2	20	1	10	6	60	3	30	4	40
Grade 3	0	0	0	0	0	0	2	20	0	0	6	60
p-value*	0.035**				0.002**				<0.001**			

*Nonparametric Mann Whitney U test. Leve of Confidence 95%

**Statistically significant differences; *p* < 0.05

Table 3 presents the data relative to, evaluates the evolution of remineralization of the samples within each mouthwash treatment, according to the exposure time using the Friedman statistical test. In the xylitol group, the proportion of unchanged enamel samples (Grade 0) decreased from 70% after 15 days to 40% after 30 and 45 days, with a concomitant increase in Grades 1 and 2 (p = 0.001). Similarly, fluoride treatment led to a reduction in the percentage of Grade 0 from 20% (15 days) to 0% after 30 days, while there was a progressive increase in Grades 1, 2, and 3 - in particular, Grade 3, which reached 60% at 45 days (p = 0.001).

**Table 3:** Degree of in vitro remineralization of the enamel of demineralized permanent molars, according to the time of exposure to Xylitol and Fluoride mouthwashes

Remineralization	15 days		30 days		45 days		p-value*
Xylitol	n	%	n	%	n	%	
Grade 0	7		4		4	40	0.001**
Grade 1	3		5		3	30	
Grade 2	0	0		10	3	30	
Grade 3	0		0	0	0	0	
Fluoride							
Grade 0	2	20	0	0	0	0	0.001**
Grade 1	6		2	20	0	0	
Grade 2	2		6		4	40	
Grade 3	0	0	2	20		60	

* Friedman test. Leve of Confidence 95%

**Statistically significant differences; *p* < 0.05

Table 4 shows the data obtained by the post hoc multiple comparisons (Hom test) to determine at what times the temporal differences were significant. For xylitol, treatment, significant differences were found between 15 and 30 days (p = 0.038), 15 and 45 days (p = 0.031) and 30 and 45 days (p = 0.038), indicating continuous improvement over time. In contrast, fluoride treatment showed a significant difference only between 15 and 45 days (p = 0.049), with no relevant variation between 15-30 days (p = 0.346) or between 30-45 days (p = 0.144), indicating that its maximum effect would be reached at the end of the time interval evaluated.

**Table 4:** Multiple comparisons of the effects of the time of exposure of demineralized permanent molar to xylitol and fluoride containing mouthwashes, on their *in vitro* remineralization

Mouthwash	Time	p-value*	
		45 days	30 days
Xylitol	15 days	*p* = 0.031**	*p* = 0.038**
	30 days	*p* = 0.038**	
Fluoride	15 days	*p* = 0.049**	*p* = 0.144
	30 days	*p* = 0.346	

*Holm post hoc test for Friedman multiple comparisons

**Statistically significant differences; *p* < 0.05

**Figure 1:**
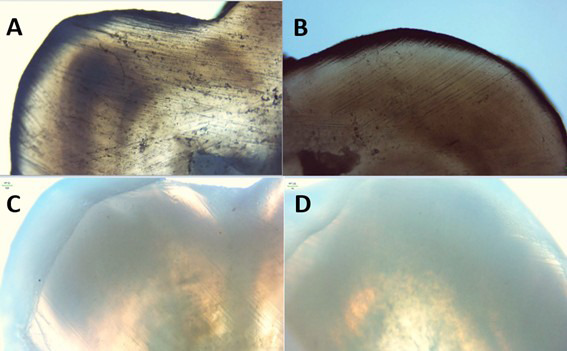
*In vitro* remineralizing effect of xylitol rinsing on an enamel sample. A. Negative control B. Degree of remineralization “0” after time interval of 15 days. C. Grade “1” Remineralization at 30 days. D. Grade “1” Mineralization at 45days.

**Figure 2:**
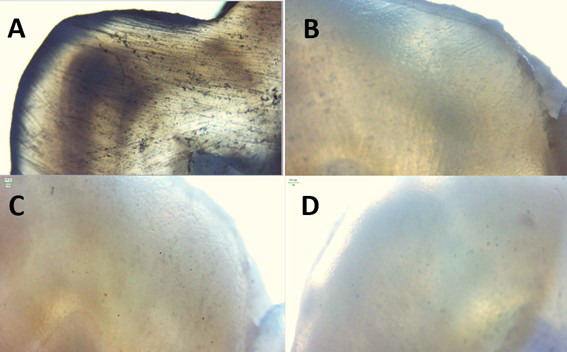
*In vitro* remineralizing effect of fluoride mouthwash on an enamel sample A. Negative control B. Grade “1” Remineralization g after 15 days. C. Grade “2” Remineralization after 30 days. D. Grade “3” Remineralization after 45 days.

## DISCUSSION

Dental remineralization is a process that can occur naturally or be stimulated through specific treatments. It consists of reintroducing minerals from saliva and the biofilm surrounding the tooth to areas that have suffered a partial loss of minerals (demineralization). Among the therapeutic options, the use of fluoride is the type that has received the greatest scientific support ^15^. In addition to fluoride therapies, there has been growing interest in fluoride-free alternatives. These compounds, of biological origin and generally extracted from natural products, such as Xylitol, are being evaluated as possible therapeutic options ^5^.

The results of this study confirmed that mouthwashes containing fluoride and xylitol have a remineralizing effect on demineralized permanent molars, which is consistent with previous studies. Researchers such as Kilic et al. ^16^ have also concluded that the fluoride-free xylitol remineralizing agent had the lowest Ca/P ratio compared with the other remineralizing agents studied. In contrast, Jiménez et al. ^5^ have discovered that Xylitol showed greater remineralizing properties when compared with the other experimental groups. The findings of this study suggested that both mouthwashes offered clinical benefits, although fluoride appeared to be more effective in direct remineralization. 

Although the mouthwash with Xylitol, showed a lower remineralizing effect when er compared with that of Fluoride, the results indicated an improvement in demineralized enamel surfaces. Jiménez et al. ^5^, presented results that differ from those of our study, as they found that the paste with Xylitol showed greater remineralizing properties in comparison with other experimental groups. These differences could be due to the use of a different experimental model that uses a cyclic pH model to simulate the oral environment, thus showing a higher remineralizing property of Xylitol by means of energy dispersive spectroscopy (EDS) and scanning electron microscopy (SEM) analyses, when compared with other experimental groups. This could explain the variation in the results since Xylitol seems to interact in solution with calcium and phosphate, acting as an essential transporter for enamel remineralization ^17^.

With regard to exposure time, within the present study, we can say that after 45 days, both the mouthwashes with Xylitol and that with Fluoride were effective in remineralizing enamel. This finding allows us to understand that Xylitol and Fluoride may have a threshold of effectiveness in remineralization depending on time, and that this must be respected to maximize their benefits. The above-mentioned findings are in agreement with those of Cobos et al. ^13^. Who determined that in the 45-day group, in all cases, there was at least one change in the enamel surface. Moreover, this was not limited only to the surface, but a modification in the subsurface characteristics was also observed. 

One of the main limitations of this study was the relatively short duration of the mouthwash application time interval, which could have limited the extent of the remineralizing effect observed. Furthermore, the number of samples was limited, which could have influenced the representativeness of the results. An important to consider is that the controlled environment in which the experiment was conducted does not fully reflect real-life oral conditions, such as the presence of biofilm or changes in oral pH, factors that could have altered the results obtained. While the results confirmed the potential of Fluoride as a remineralizer par excellence, it is also important to mention the potential of Xylitol, which could be used as a complement in those patients seeking an additional approach to bacterial control, but not as a primary treatment for remineralization.

## CONCLUSIONS

The results of this in vitro study demonstrate that both xylitol and fluoride mouthwashes have a remineralizing effect on the enamel of demineralized permanent molars. A statistically significant difference was; however, observed in favor of the fluoride mouthwash, which exhibited a higher degree of remineralization. Furthermore, exposure period of 45 days was determined as being the optimal interval for both formulations to maximize mineral recovery in the tooth enamel.

## Source of financing:

Self-financed.
